# Supplementary material for: Accuracy and precision of citizen scientist animal counts from drone imagery
Source: PLoS One. 2021 Feb 22;16(2):e0244040. doi: 10.1371/journal.pone.0244040 (PMC7899343; doi:10.1371/journal.pone.0244040)
Supplement: S1 File — (DOCX) [file pone.0244040.s001.docx]

**S1 File.**

There are more than 100 different projects on Zooniverse, which has over a million participants (zooniverse.org). Each project on Zooniverse has a unique URL and customizable tabs. Data are collected under the Classify tab, where multiple workflows can be input and tailored to the goal of the project. Each project must also create a detailed tutorial that volunteers read before participating. For our tutorial, we used flipper orientation as a guide for counters instead of size or coloration. The relative size of seals versus sea lions was not a reliable identification factor since all age classes of seals and sea lions can be found on Año Nuevo Island. For example, an adult female sea lion could be confused for a juvenile elephant seal if identification is based solely off size. The color was also unreliable because a molting seal can closely match a sea lion's brown color.

Zooniverse contains many areas for volunteers to learn about the project. Each site can create a field guide, where individual species are highlighted, and photographs can be uploaded. We included extra information about seals, sea lions, pelicans, cormorants, and gulls in our field guide. Under the "About" tab on the project homepage, volunteers can read more about the project, meet team members, see preliminary results (if there are any) and browse some frequently asked questions. We made sure each of these sections was filled out and up to date throughout the project. Zooniverse also provides Talk Boards for each project, where project managers and volunteers can interact. When viewing photographs, volunteers can share them directly to the talk boards with questions or comments.

After our initial launch, we planned various events and announcements to keep volunteers engaged and return to the project. We tailored our outreach to groups already established in the marine science field. For example, we reached out to local organizations such as The Marine Mammal Center, the Seymour Marine Discovery Center, and Año Nuevo State Park. These volunteers often had a vested interest in the project and shared the project with visitors at their respective establishments. We also held physical events in Santa Cruz, some organized by ourselves and others, such as Citizen Science night at the Santa Cruz Public Library and Science Sunday at the Seymour Center. We gave middle school and high school class presentations, where students could learn about the scientific process and participate by counting animals themselves.

On the website, we regularly updated information, especially on the talk boards. We utilized announcement banners for larger events and created "hashtags" such as #cormorantcount, where volunteers shared how many cormorants they counted in a particular photograph. By having volunteers share how many animals they counted using hashtags, our goal was to create a sense of community and increase talk board participation. Email newsletters were sent roughly once a month to volunteers with information about preliminary results, events, and contests. We hosted two counting contests throughout the project. Both contests had prizes (stickers and stuffed animals) for the three volunteers that counted the most photographs over a week.

**S1 Table**. Metadata for drone flights with expert adult and pup counts for each flight.

| Date | Total Image Tiles | Total elephant seals | Number of Adults | Number of Pups |
| --- | --- | --- | --- | --- |
| 2017-07-15 | 830 | 128 | 128 | 0 |
| 2017-07-22 | 742 | 72 | 72 | 0 |
| 2017-08-02 | 731 | 83 | 83 | 0 |
| 2017-08-07 | 753 | 63 | 63 | 0 |
| 2017-08-16 | 717 | 34 | 34 | 0 |
| 2017-08-23 | 796 | 38 | 38 | 0 |
| 2017-09-01 | 707 | 58 | 58 | 0 |
| 2017-09-11 | 774 | 104 | 104 | 0 |
| 2017-09-23 | 746 | 259 | 259 | 0 |
| 2017-10-03 | 679 | 334 | 334 | 0 |
| 2017-10-10 | 700 | 325 | 325 | 0 |
| 2017-10-22 | 700 | 341 | 341 | 0 |
| 2017-10-31 | 703 | 339 | 339 | 0 |
| 2017-11-18 | 701 | 350 | 350 | 0 |
| 2017-11-29 | 642 | 256 | 256 | 0 |
| 2017-12-07 | 800 | 294 | 294 | 0 |
| 2017-12-18 | 720 | 204 | 204 | 0 |
| 2018-01-13 | 664 | 590 | 411 | 179 |
| 2018-01-23 | 719 | 794 | 509 | 285 |
| 2018-02-07 | 797 | 785 | 429 | 356 |
| 2018-02-13 | 584 | 608 | 276 | 332 |
| 2018-02-28 | 622 | 469 | 76 | 393 |
| 2018-03-06 | 775 | 462 | 85 | 377 |
| 2018-03-18 | 797 | 358 | 100 | 258 |
| 2018-04-01 | 798 | 649 | 335 | 314 |
| 2018-04-17 | 801 | 744 | 590 | 154 |
| 2018-04-27 | 686 | 1071 | 867 | 204 |
| 2018-05-03 | 848 | 1200 | 1054 | 146 |
| 2018-05-22 | 1259 | 1155 | 925 | 230 |
| 2018-06-17 | 745 | 248 | 248 | 0 |
| 2018-06-26 | 770 | 122 | 122 | 0 |
| 2018-07-17 | 945 | 93 | 93 | 0 |
| 2018-08-12 | 827 | 57 | 57 | 0 |
| 2018-08-25 | 770 | 35 | 35 | 0 |
| 2018-09-12 | 802 | 89 | 89 | 0 |
| 2018-10-08 | 655 | 126 | 126 | 0 |
| 2018-10-20 | 727 | 195 | 195 | 0 |
| 2018-10-28 | 735 | 193 | 193 | 0 |
| 2018-12-06 | 786 | 248 | 248 | 0 |
| 2018-12-15 | 738 | 208 | 208 | 0 |
| 2018-12-22 | 622 | 133 | 133 | 0 |
| 2019-01-02 | 732 | 231 | 198 | 33 |
| 2019-02-23 | 873 | 499 | 134 | 365 |
| 2019-03-08 | 863 | 371 | 95 | 276 |
| 2019-05-07 | 870 | 1145 | 1145 | 0 |
| 2019-05-18 | 922 | 1306 | 1306 | 0 |
| 2019-06-07 | 986 | 592 | 592 | 0 |
| 2019-06-22 | 952 | 166 | 166 | 0 |
| 2019-07-05 | 882 | 81 | 81 | 0 |
| 2019-07-17 | 949 | 80 | 80 | 0 |
| 2019-07-25 | 1036 | 65 | 65 | 0 |

**S2 Table.** Species abundance using several median algorithms and their associated percent error. For the expert seal count, only elephant seals (*M. angustirostris*) were included. For expert sea lion counts, Steller sea lions (*E. jubatus*) and California sea lions (*Z. californianus*) were counted separately but combined to compare with citizen science counts, who differentiated between families but not species (family *Phocidae*: *M. angustirostris and P. vitulina*; family *Otariidae*: *E. jubatus, Z. californianus*).

| Species | Date | Expert Count | Median Citizen Science Count | Median[3:Max] Citizen Science Count | Median[1:Max-2] Citizen Science Count | % error in Median | % error in Median[3:Max] | % error in Median[1:Max-2] |
| --- | --- | --- | --- | --- | --- | --- | --- | --- |
| Seals | 2017-07-15 | 217 | 157 | 335 | 71 | 28% | 54% | 67% |
| Seals | 2018-01-23 | 789 | 610 | 834 | 463 | 23% | 6% | 41% |
| Seals | 2018-06-26 | 161 | 121 | 273 | 69 | 25% | 69% | 57% |
| Seals | 2019-02-23 | 489 | 364 | 521 | 260 | 26% | 6% | 47% |
| Seals | 2019-07-05 | 136 | 80 | 266 | 33 | 42% | 95% | 76% |
| Sea lions | 2017-07-15 | 5469 | 4212 | 5253 | 3403 | 23% | 4% | 38% |
| Sea lions | 2018-06-26 | 3525 | 2561 | 3131 | 1953 | 27% | 11% | 45% |
| Sea lions | 2019-07-05 | 4663 | 3329 | 4058 | 2334 | 29% | 13% | 50% |

­­­


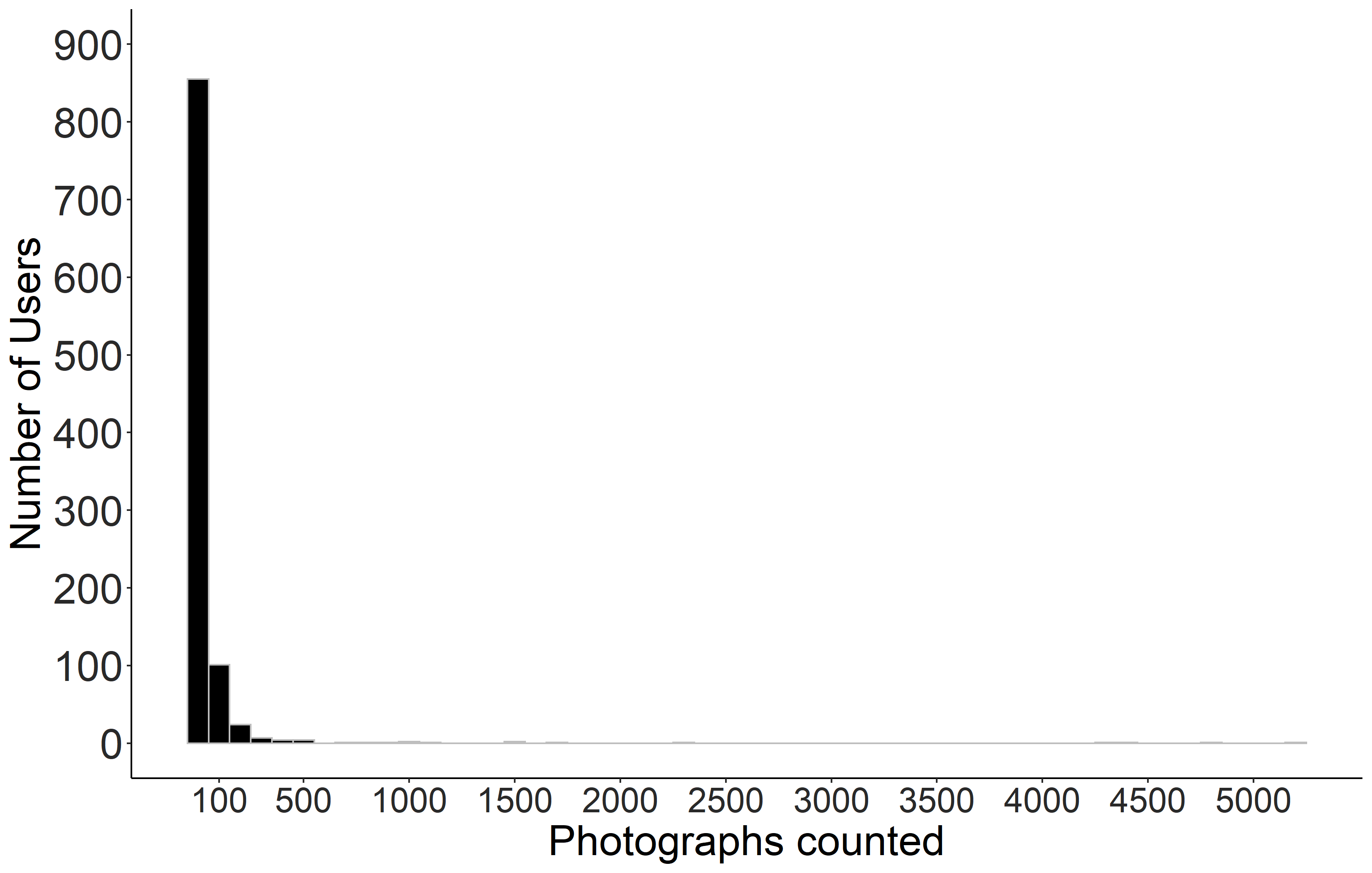


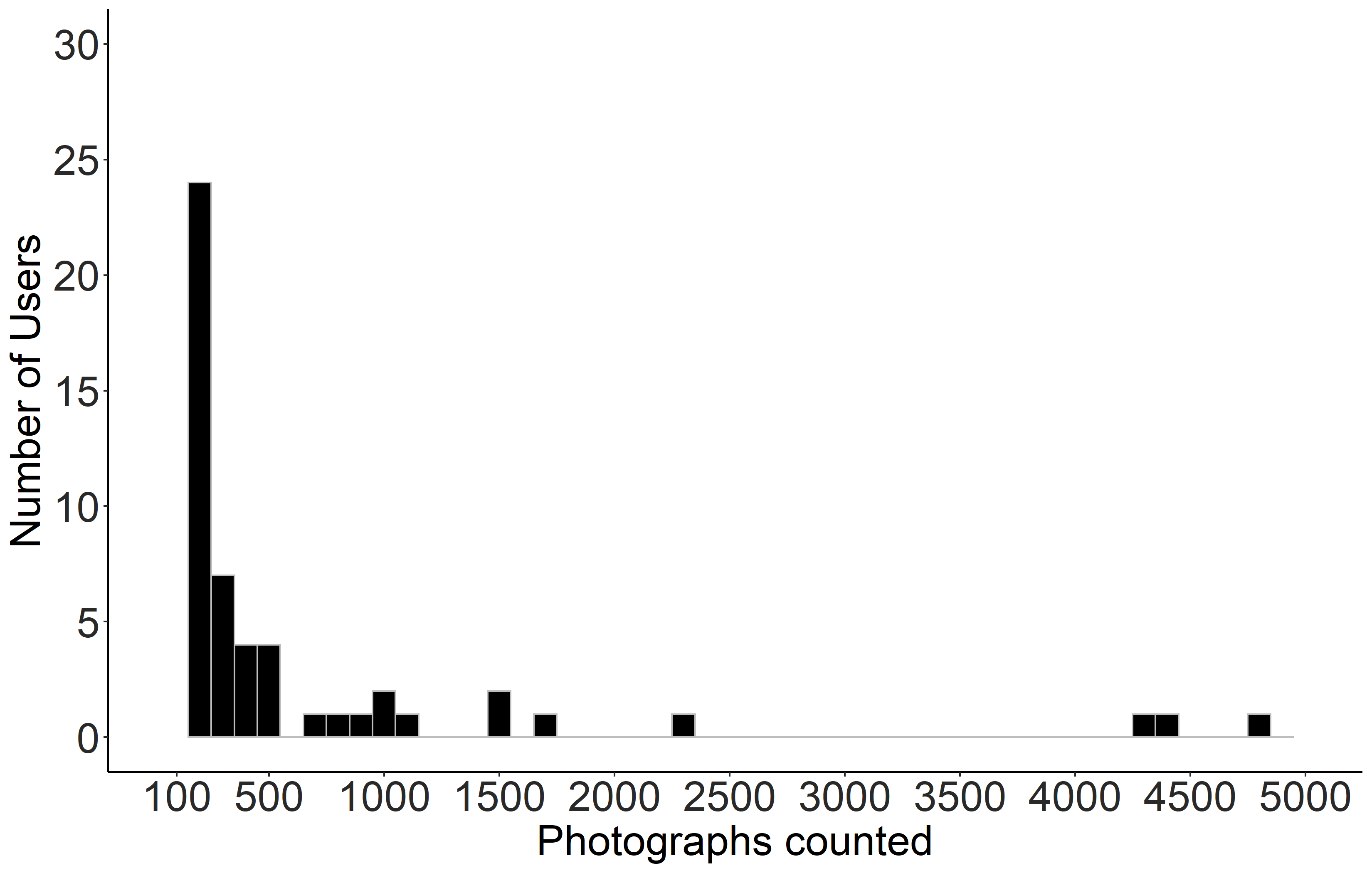


**S1 Fig.** Comparing volunteer participation in the project. Photographs counted by individuals not signed into their accounts are not included. Most users counted 1-100 photographs (top). Below, volunteers counting 1-100 photographs are excluded to visualize the spread of data better. 62 volunteers counted 100-500 images. 18 volunteers counted above 500 images, with a maximum of 5,153 photographs counted.


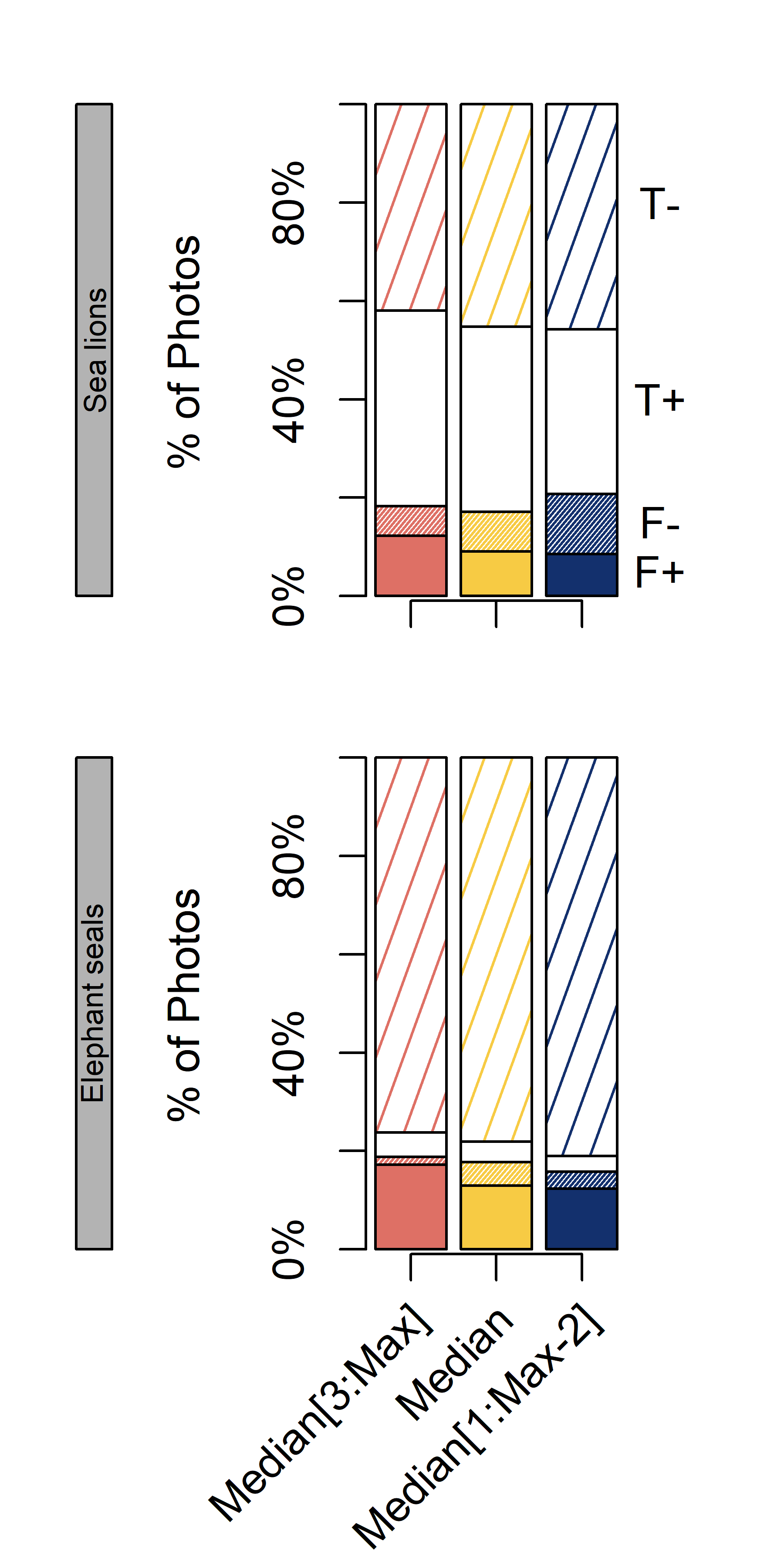


**S2 Fig**. The proportion of sea lion and elephant seal photographs classified accurately (T- true negative when citizen scientists correctly identified no animals in a photograph or T+ true positive when citizen scientists correctly identified existing animals in a photograph) or inaccurately (F- false negative when citizen scientists incorrectly indicated that there were no animals in a photograph or F+ false positive when citizen scientists incorrectly indicated that there were animals in a photograph).
